# Pulsatile tinnitus: treatment with clonazepam and propranolol

**DOI:** 10.1016/S1808-8694(15)31297-0

**Published:** 2015-10-20

**Authors:** Sergio Albertino, Aída R.M. de Assunção, Jano A. Souza

**Affiliations:** 1 Joint Professor, Universidade Federal Fluminense; 2 Assistant Professor, Universidade do Estado do Rio de Janeiro; 3 Ph.D. in Neurology, Universidade Federal Fluminense

**Keywords:** pulsatile tinnitus, vascular disease, treatment

## Abstract

Pulsatile tinnitus synchronous with heartbeat is rare and normally has vascular origin: arterial (malformation, arterial anatomical variation) or venous (aberrant jugular bulb, glomus tumors, tympanic glomus tumor). Early etiology identification is essential for appropriate treatment to be established. Magnetic angioresonance makes the vascular identification possible and precise. We report a case of arterial anatomical variation in which the treatment was propranolol and clonazepam, showing tinnitus improvement.

## INTRODUCTION

Tinnitus is a frequent complaint in the office of otorhinolaryngologists and neurologists, but pulsatile tinnitus synchronous with heartbeat is less frequent and may be caused by different arterial or venous vascular etiologies. Identification of the etiology of this type of tinnitus is essential to define the appropriate therapy and to prevent sequelae that may result from late or incorrect diagnosis. Arterial pulsatile tinnitus may result from malformations of arterial-venous fistulas, atherosclerosis, vascular stenosis, ectopic intratympanic carotid artery, persistence of stapedial artery, stria vascularis aberrant artery, high cardiac output (anemia, thyrotoxicosis). Venous pulsatile tinnitus may be primary (jugular bulb abnormalities, jugular or tympanic glomus tumor) or secondary to intracranial hypertension.

The purpose of the present study was to report a case of tinnitus of arterial vascular etiology that was difficult to solve and the approach followed to reach relief of tinnitus.

## CASE REPORT

Male 51-year-old patient without systemic or otological disease, complained of tinnitus of pulsatile nature on the left, synchronous with heartbeat, which had started 9 months before and hindered his professional performance (administrative assistant) and resulted in marked anxiety. The patient had taken antidepressants, anxiolitic and vasoactive drugs prescribed by the neurology, without any response. ENT physical examination presented normal otoscopy and the patient had past history of septoplasty two years before the onset of tinnitus. Neurological examination had no significant findings.

**Figure 2:**
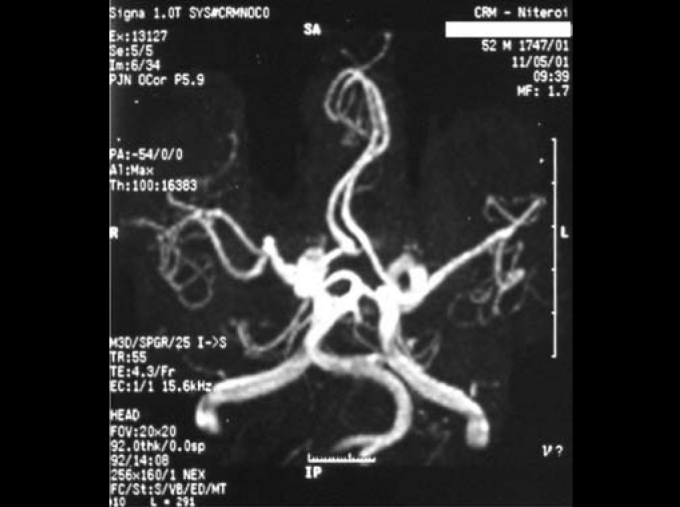
Tortuous vertebral-basilar transition.

We conducted the following complementary tests:

– Pure tone audiometry – mild decrease in high frequencies (3000Hz 30 dB; 4000Hz 35 dB; 6000Hz 40 dB) bilaterally.
– Brainstem evoked potential did not evidence any abnormalities.
– Nuclear Magnetic Resonance of the head, with gadolinium– isolated focus of reduced signal in T1 and elevated in T2, not impregnated by the contrast medium in the left frontal radial crown, suggestive of cerebral lacuna. Basilar artery was elongated and tortuous, describing anterior-lateral loop on the left under the pons.
– Head angioresonance - brain images in T2 showed hyperintense focus on left radial crown, which may correspond to small area of gliosis or lacuna infarction. Tortuous vertebral-basilar transition towards the pontine-cerebellum angle cistern on the left (Figure 2).

In both neuroimaging studies the other head and vascular structures were normal.

After excluding the hypothesis of surgical treatment, we prescribed clonazepam 2mg/day associated with propranolol 20mg TID, which made tinnitus more tolerable to the patient.

## DISCUSSION

**Figure 1:**
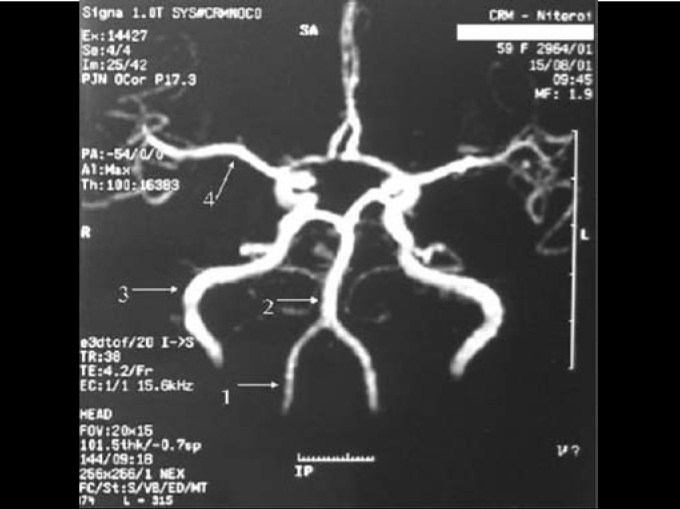
Normal anatomy of arteries: 1 – vertebral; 2 – basilar; 3 – internal carotid; 4 – media cerebral artery.

Propranolol is an example of a typical beta-blocker. Its main effects are reduction in heart rate and myocardial contractility, reduction of systolic volume, reduction of cardiac output and reduction of arterial blood pressure. Pulsatile tinnitus may be generated by blood flow turbulence caused by vascular malformations that affect vessel lumen, as observed by comparing the normal anatomy of vertebral-basilar system (Figure 1) with the malformation presented by the patient (Figure 2). The use of a beta-blocker aimed at reducing heart rate and arterial flow, maintaining stable blood pressure and reducing perception of tinnitus. The association with clonazepam (long-action benzodiazepine, frequently used to treat tinnitus) was made because of its anxiolitic and myorelaxing effect and it proved to be quite effective with good control of patients' symptomatology.

## CLOSING REMARKS

Unilateral pulsatile tinnitus is not normally followed by specific audiometric alterations and should be investigated by neuroimaging exams to be properly diagnosed. In this case there was no indication for surgical intervention and we decided to prescribe benzodiazepine agent associated with beta-blocker to reduce the perception of tinnitus and heart rate allowing the patient to carry on with his daily activities.

## References

[1] Albertino S, Moreira Filho PF. (2000). Benzodiazepínicos: Atualidades.. RBM-ORL.

[2] Fukuda Y. (1997). Zumbido: diagnóstico e tratamento.. RBM-ORL.

[3] Guimarães AC., Silva P. (1980). Farmacologia..

[4] Kauffman EA, Nadaf LC, Souza RT. (2001). Diagnóstico diferencial e conduta no zumbido pulsátil.. RBORL.

[5] Moller AR., Jackler RK., Brackmann DE. (1994). Neurotology..

[6] Sanchez TG, Murao MS, Miranda IRT, Kii MA, Bento RF, Caldas JG (2001). Uma nova terapêutica para o tratamento do zumbido pulsátil objetivo de origem venosa.. Arquivos da Fundaçã o Otorrinolaringologia.

[7] Yoo TJ, Shulman A, Brummett RE, Griest SE, Mulkey M, Rubenstein M., Shulman A, Aran JM, Tonndorf J, Feldmann H, Vernon JA. (1997). Tinnitus diagnosis/treatment..

